# Tissue-specific *NIA1* and *NIA2* expression in *Arabidopsis thaliana*

**DOI:** 10.1080/15592324.2019.1656035

**Published:** 2019-08-22

**Authors:** Justyna J. Olas, Vanessa Wahl

**Affiliations:** a Department of Metabolic Networks, Max Planck Institute of Molecular Plant Physiology, Potsdam, Germany; b Department of Molecular Biology, University of Potsdam, Institute of Biochemistry and Biology, Potsdam-Golm, Germany

**Keywords:** Arabidopsis, NIA1, NIA2, nitrate assimilation, plant development, RNA in situ hybridization, expression, cell- and tissue-specificity

## Abstract

Nitrogen (N) is an essential macronutrient for optimal plant growth and ultimately for crop productivity Nitrate serves as the main N source for most plants. Although it seems a well-established fact that nitrate concentration affects flowering, its molecular mode of action in flowering time regulation was poorly understood. We recently found how nitrate, present at the shoot apical meristem (SAM), controls flowering time In this short communication, we present data on the tissue-specific expression patterns of *NITRATE REDUCTASE 1 (NIA1)* and *NIA2 in planta*. We show that transcripts of both genes are present throughout the life cycle of *Arabidopsis thaliana* plants with *NIA1* being predominantly active in leaves and *NIA2* in meristematic tissues.

## Introduction

In *Arabidopsis thaliana* (Arabidopsis), nitrate, once taken up by roots, is distributed to sink tissues including meristems via xylem vessels.^1^ It is assimilated into ammonia by nitrate reductase, encoded by *NITRATE REDUCTASE 1* (*NIA1*) and *NIA2*, and further converted into amino acids,^2^ which serve to support biological processes. Our previous analyses showed that nitrate is present at the shoot apical meristem (SAM) of Arabidopsis plants, where it directly triggers flowering.^3^ We demonstrated that a moderate, non-stressful restriction of nitrate availability delays flowering time, due to decreased expression of *SUPRESSOR OF OVEREXPRESSION OF CONSTANS 1* (*SOC1*) and *SQUAMOSA PROMOTER BINDING PROTEIN-LIKE3* (*SPL3*) and *SPL5* at the SAM. Moreover, we found that the nitrate-inducible genes *NIA1* and *NIA2* and the master regulators of nitrate signaling, *NIN-LIKE PROTEIN 6* (*NLP6*) and *NLP7*, ^4^ are present at the SAM. We concluded that nitrate controls the flowering time at the SAM through NLP6 and NLP7 by regulating *SOC1* at least in part via *SPL3* and *SPL5*.^3^

Interestingly, we found that *NIA1* and *NIA2* expression patterns at the SAM were barely overlapping with each other, suggesting that they might be regulated independently and assimilate nitrate in a stage- and tissue-dependent manner. Since their transcriptional response rapidly and directly responds to nitrate and does not require *de novo* protein synthesis, ^5^ the analysis of *NIA* transcripts provides information on the status of nitrate signaling and assimilation in cells. Although the biological function of *NIA1* and *NIA2* [e.g. 6] and nitrate-dependent expression of promoter-reporter constructs^7,8^ were previously studied, we provide first tissue- and cell-specific expression analyses for the first step of nitrate assimilation *in planta*.

## Results

**Figure 1.**
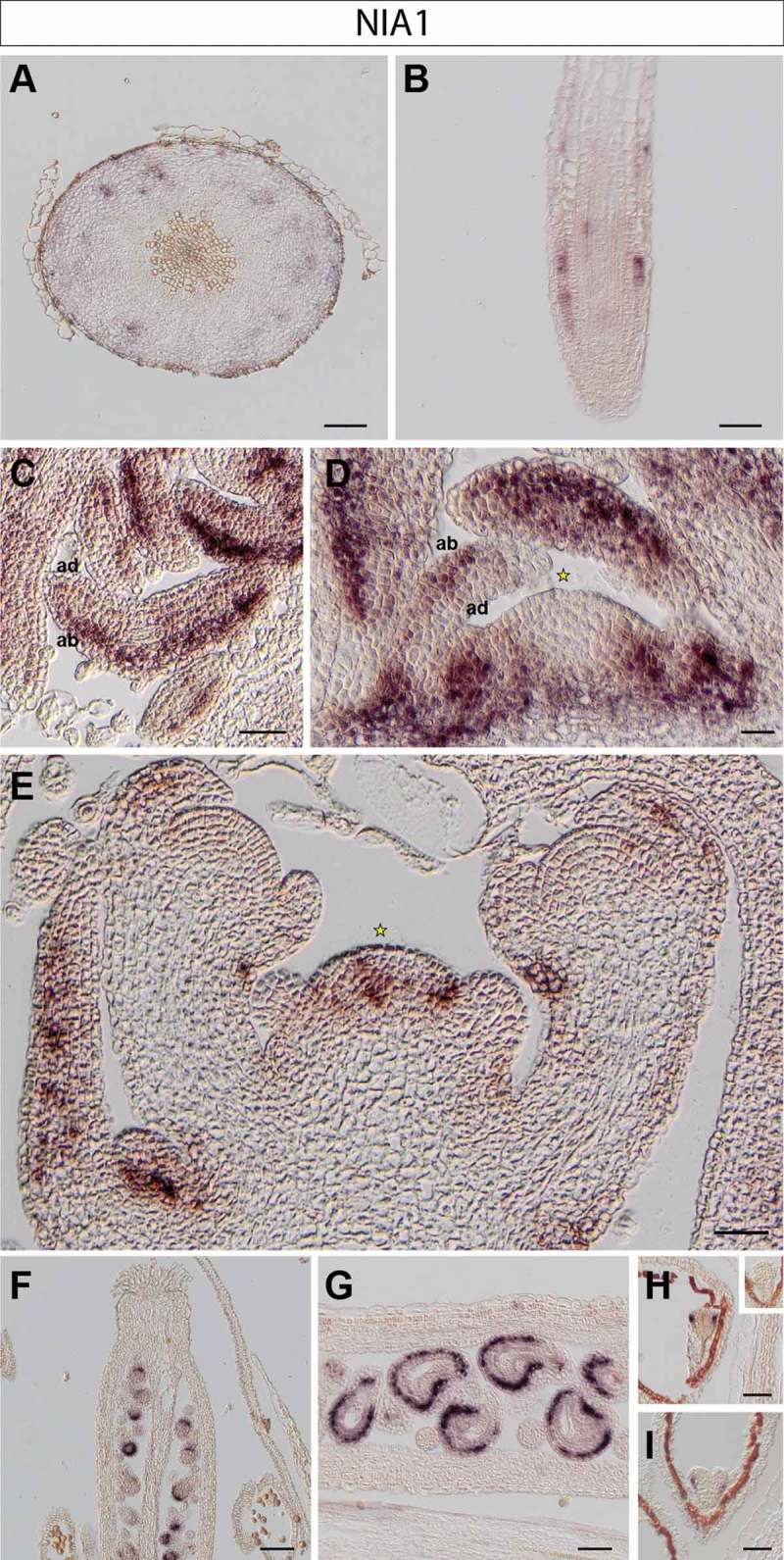
NIA1 expression in Arabidopsis thaliana plants. RNA in situ hybridization using a specific probe for Arabidopsis NIA1 on transversal sections through the hypocotyl (A), longitudinal sections through a root tip (B), rosette leaves (C), the vegetative shoot apical meristem (D), the inflorescence meristem (E) and young pistils, containing ovules (F-G). Representative stains in developing embryos (H-I): early (H) and late heart stages (I). Inset in H: globular stage embryo. Scale bars 20µm (A-D, F-I) and 50µm (E).

**Figure 2.**
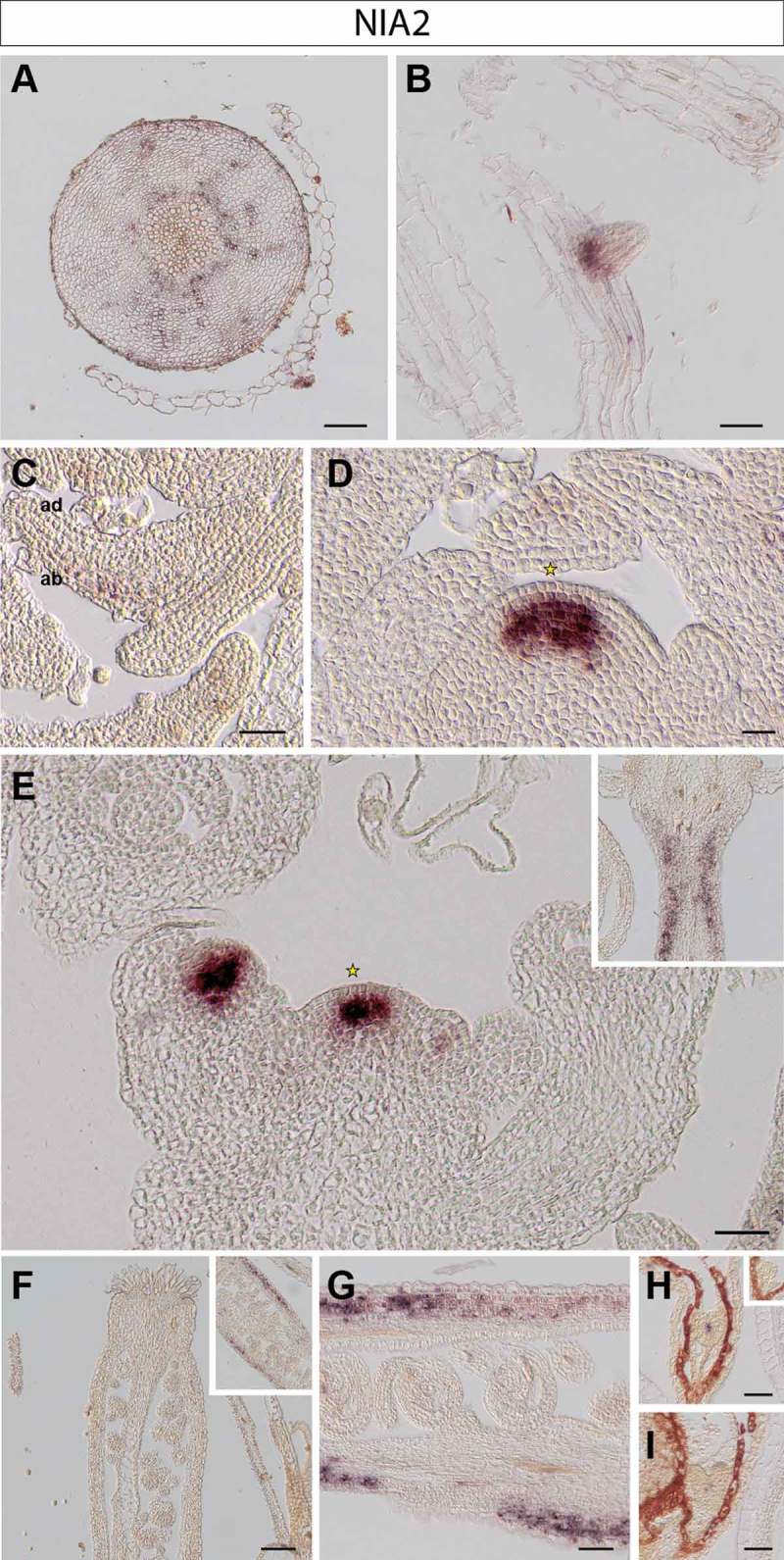
NIA2 expression in Arabidopsis thaliana plants. RNA in situ hybridization using a specific probe for Arabidopsis NIA2 on transversal sections through the hypocotyl (A), longitudinal sections through an emerging lateral root tip (B), rosette leaves (C), the vegetative shoot apical meristem (D), the inflorescence meristem (E) and various stages of pistil development (F-G). Inset in E: replum and upper part of pedicel. Representative stains in developing embryos (H-I): early (H) and late heart stages (I). Inset in H: globular stage embryo. Scale bars 20µm (A-D, F-I) and 50µm (E).

We made use of RNA *in situ* hybridizations, in order to investigate cell- and tissue-specific expression of *NIA1* and *NIA2* transcripts *in planta*. Using tissue-specific probes for *NIA1* and *NIA2*,^3^ we detected transcripts in various tissues of Arabidopsis wild-type plants. Representative images of different tissue sections are presented in Figures 1 and 2.

In the hypocotyl, *NIA1* is present in distal phloem cells but absent from proximal tissue including phloem, cambium and xylem cells (Figure 1(a)). In contrast, *NIA2* is expressed throughout the cambium (likely including the bifacial stem cells and distal cambium) and phloem cells throughout a cross section of the hypocotyl (Figure 2(a)).

In roots, *NIA1* is expressed in epidermis cells of the root meristem, with some weaker expression in vascular layers, presumably in phloem cells (Figure 1(b)). We did not observe any *NIA2* expression in main root meristematic zones (not shown); however, transcript of *NIA2* is found at the base of newly emerging lateral root primordia, coinciding with procambium and young vasculature cells^9^ (Figure 2(b)).

We found that *NIA1* is predominantly present in all leaf stages, including newly formed leaf primordia, spreading from the proximal to the distal part of young leaves. Its expression domain is, however, restricted to the abaxial side, which corresponds to spongy mesophyll cells^10^ (Figure 1(c,d)). Instead, transcript of *NIA2* is barely detectable in leaves and fully absent from young leaf primordia (Figure 2(c,d)).

*NIA1* transcript is also present in cauline leaves of the inflorescence apex (Figure 1(e)). Moreover, as previously observed,^3^ *NIA1* is expressed only in the rib and peripheral zones of the SAM and axillary meristems, but is not present in the central region including the organizing center and stem cell niche of the vegetative (Figure 1(d)) and the inflorescence SAM (Figure 1(e)). *NIA2* is strongly expressed in the center of the vegetative (Figure 2(d)), inflorescence and floral meristems (Figure 2(e)).^3^ Starting in L3 the *NIA2* expression domain overlaps with the organizing center of the SAM and the region lacking *NIA1* transcript (compare Figures 1(d) and 2(d)).

As different expression patterns of *NIA*s are observed in floral meristems, we also analyzed their expression in crucial stages of seed development. Expression of *NIA1* is found in the cells of the inner integument in ovules (Figure 1(f,g)), where *NIA2* is not detectable (Figure 2(f,g)). In contrast, the signal of *NIA2* is found in valves of siliques (Figure 2(g)).

After fertilization, *NIA1* is expressed in the various stages of embryo development. We found that *NIA1* expression is more abundant at the abaxial side of newly formed cotyledons in early heart stage embryos^11^ (Figure 1(h)). In later heart stage embryos, expression of *NIA1* is present in protodermal cells in the basal portion of the embryo,^12^ in the region destined to give rise to the hypocotyl (Figure 1(i)). *NIA1* transcript is not detectable in globular (inset of Figure 1(h)) or mature embryos (not shown). In contrast, *NIA2* is expressed in procambial cells in the central zone of early and later heart stages^13^ (Figure 2(h,i)), but is absent from the globular (inset of Figure 2(h)) and more mature stages of embryogenesis (data not shown).

## Conclusions

We found that the expression pattern of *NIA1* complemented that of *NIA2* in the same organ within the set of tissue samples analyzed, suggesting that nitrate is assimilated by nitrate reductase encoded by either *NIA1* or *NIA2* in a cell- or tissue-specific manner. This further indicates non-redundant functions of the two NIAs in nitrate assimilation that might be associated with cell specification and plant development. This is in agreement with previous reports showing that single mutants of *nia1* and *nia2* showed different responses and sensitivity levels to, e.g. salicylic acid^14^ or cytokinin treatments.^15^ It should, however, be kept in mind that expression of both genes is regulated by various other endogenous and exogenous factors resulting in a diurnally differential expression pattern, ^16^ which might result in changes of expression domains not covered in this study.

*NIA1* function seems to be predominantly restricted to leaf tissues. Apart from rosette leaves, this includes cotyledon primordia, cauline leaves, and cells within organ primordia destined to become leaf-like organs, but also the inner integument of developing ovules. The establishment of the inner ovule layer is known to be the earliest morphological sign of the adaxial-abaxial polarity.^17^ The localization of *NIA1* transcript at the abaxial side of newly formed cotyledons during embryogenesis, rosette leaves, and the inner layer of the integument suggests that *NIA1* might be involved in the spatiotemporal regulation of polarity in general. *NIA2*, on the other hand, is active in highly dividing meristematic regions such as within the SAM and cambial cells. The analysis of the single and double *nia* knockout mutants would be required to elucidate the role of *NIA1* in the establishment of the abaxial-adaxial polarity and *NIA2* in the regulation of meristematic niches in plants.

In summary, our results suggest that both *NIA*s take part in other biological and developmental processes apart from their well-established metabolic function.

## Materials and methods

### Plants material and growth conditions

*Arabidopsis thaliana* accession Columbia (Col-0) was used in this study. Plants were grown in controlled growth chambers (Percival Scientific Inc., USA) or in the greenhouse at 22°C in long- (16-h light/8-h dark) or short-day (8-h light/16-h dark) photoperiods. Plant material for embedding was generally harvested toward the end of the day.

### RNA *in situ* hybridization

For RNA *in situ* hybridization apex, hypocotyl, root, silique and flower samples were harvested, fixed with FAA solution (formaldehyde, ethanol, acetic acid; ASP300S, Leica) and embedded into wax (EG1160, Leica) as previously reported.^18^ RNA *in situ* hybridization was carried out as described.^18^ Probes for *NIA1* and *NIA2* were previously published.^3^
